# Virulence and biocontrol potential of entomopathogenic nematodes against soil‐dwelling stages of the small hive beetle under laboratory and semi‐field conditions

**DOI:** 10.1002/ps.8766

**Published:** 2025-03-11

**Authors:** Sitaram Aryal, Alihan Katlav, Clarissa M. House, Robert N. Spooner‐Hart, Michael Duncan, Uffe N Nielsen, James M. Cook, Markus Riegler

**Affiliations:** ^1^ Hawkesbury Institute for the Environment, Western Sydney University Penrith Australia; ^2^ School of Science Western Sydney University Penrith Australia

**Keywords:** *Aethina tumida*, *Heterorhabditis* spp., *Steinernema* spp., Nitidulidae, virulence

## Abstract

**BACKGROUND:**

The small hive beetle (SHB; *Aethina tumida*) is a significant pest affecting honey bees and the global beekeeping industry. The harmful effects of chemical pesticides on bee health, non‐target species and ecosystems highlight the need for sustainable SHB control methods. Soil applications of entomopathogenic nematodes (EPNs) targeting the soil‐dwelling life stages (wandering larvae, pupae and emerging adults) of SHB present a promising biological control approach. We conducted comprehensive laboratory experiments to evaluate the biocontrol potential of 32 Australian isolates of five EPN species (*Heterorhabditis bacteriophora*, *Heterorhabditis indica*, *Heterorhabditis zealandica*, *Steinernema carpocapsae* and *Steinernema feltiae*) against SHB. We also performed a glasshouse experiment testing the efficacy of nine EPN isolates in soil mesocosms that simulated field conditions.

**RESULTS:**

We demonstrated that all isolates caused mortality in all life stages, with wandering larvae the most susceptible, followed by pupae and adults. Notably, *H. indica* Hi.HRN caused the highest SHB mortality, while *S. feltiae* Sf.EG was the least effective. These isolates significantly reduced SHB adult emergence, ranging from 9%–93% in autoclaved sterile soil and 16%–59% in natural soil, suggesting interaction with other soil biota. The isolates *H. indica* Hi.HRN, Hi.LMBT and *H. bacteriophora* Hb.HIE2 were the most promising candidates for biocontrol, causing >90% corrected SHB mortality in sterile soil and >80% in natural soil. Additionally, soil application of Hi.HRN caused 33% and 43% mortality of SHB adults after their emergence from natural and sterile soils.

**CONCLUSION:**

The *H. indica* isolates Hi.HRN and Hi.LMBT displayed strong biocontrol potential, warranting further evaluation. © 2025 The Author(s). *Pest Management Science* published by John Wiley & Sons Ltd on behalf of Society of Chemical Industry.

## Graphical abstract

Entomopathogenic nematodes (EPNs) are virulent against multiple life stages of *Aethina tumida* in the laboratory. EPNs significantly reduce adult emergence in natural and autoclaved sterile soil under semi‐field glasshouse conditions.

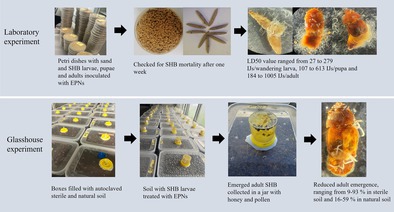

## 1 INTRODUCTION

The western or European honey bee, *Apis mellifera* Linnaeus, is the most significant pollinator of agricultural and horticultural crops globally. Originally native to Eurasia, its geographical distribution now spans every continent except Antarctica.^1^ Beyond its crucial role in pollination, the honey bee contributes significantly to the economy through the production of valuable hive products, including honey, beeswax, propolis and royal jelly.^2^ However, honey bee colonies are threatened worldwide by various factors, including pests and diseases,^3^, ^4^, ^5^ chemical pesticides,^6^ poor nutrition,^7^ habitat deterioration and climate change, along with extreme weather events.^8^ These threats collectively jeopardize the survival of honey bee colonies, placing their essential pollination services and hive product yields at risk.^9^, ^10^, ^11^ Among these threats, the small hive beetle (SHB), *Aethina tumida* Murray (Coleoptera: Nitidulidae), is a particularly significant challenge due to its serious impact on honey bee colonies and hive resources in many parts of the world where it has been established as an invasive pest originating from sub‐Saharan Africa.^12^ In Australia, it was first recorded in the Sydney region in 2002.^13^ It has since been detected in all Australian states and territories (including the incursion of a single adult SHB in the Northern Territory in September 2010 without any subsequent detections, and the detection of SHB in Tasmania in March 2023 triggering an emergency response and the declaration of successful eradication in April 2024), and is most abundant in coastal areas of New South Wales and Queensland where it causes substantial losses to beekeepers.^14^ It was estimated that damages due to SHB in Queensland were in excess of Australian $8 million from 2008 to 2011.^15^ This compares to annual losses due to SHB in the USA of US $3 million as estimated in 2004.^16^

After pupation, adults of SHB emerge from the soil and fly in search of bee colonies, where mature females lay eggs in clusters in the wax frames.^17^ Upon hatching, SHB larvae can weaken the hive by voraciously feeding on resources like pollen, honey, wax and even honey bee brood; the tunnelling of larvae through comb with stored honey and pollen further damage capped honey and comb.^12^ In addition, all motile stages can spread a yeast, *Kodamaea ohmeri*, that contributes to the fermentation of hive content.^18^, ^19^ Heavily infested colonies can suffer from slime‐out and consequently die or abscond.^12^ Adult SHB can also spread bee viruses,^20^, ^21^ further compromising hive health, and increasing colony susceptibility to parasites such as *Varroa* mites, as well as other pathogens, ultimately leading to rapid colony collapse.^22^ SHB can also attack stingless bee colonies, posing a significant threat to meliponiculture.^23^

Effective management options are necessary to mitigate the impact of SHB on apiculture. Treatment with chemical insecticides is the conventional method for controlling this pest.^24^ However, there are growing concerns about the detrimental impacts of chemical insecticides on honey bees and other beneficials, as well as on human health and the overall ecosystem.^6^, ^25^ Besides direct exposure risks, contamination of bee products with insecticides and other chemicals can be fatal to honey bees, and also affect human health.^26^, ^27^ Additionally, some populations of *A. tumida* have evolved resistance to a variety of insecticides.^28^ Hence, there is a need for sustainable alternative control measures.

SHB management methods other than chemical control involve traps,^29^, ^30^, ^31^ modified hive entrances,^32^ hive nutrient supplements,^33^, ^34^ and removal and destruction of dead colonies to prevent SHB reproduction.^35^ Moreover, several microbiological control agents have been tested to reduce the impact of SHB, including entomopathogenic fungi such as *Metarhizium* and *Beauveria*,^36^, ^37^, ^38^ and bacteria such as *Bacillus thuringiensis*.^39^ However, information on the effectiveness of these microbiological control agents remains limited, and the impact of other potential biocontrol agents needs further investigation.

As SHB individuals reach the final larval stage, they leave the hive as wandering larvae, burrowing into the soil at a depth of <20 cm and forming cells where they pupate.^40^, ^41^ Subsequently, teneral adults emerge from the subterranean pupae and need to resurface before locating a honey bee colony. This presents an opportunity to control SHB outside the hive and in the soil by targeting wandering larvae, pupae and emerging adults before they enter honey bee hives. An effective soil‐borne biocontrol agent is entomopathogenic nematodes (EPNs), which are widely used against insect pests with soil‐associated developmental stages^42^ and are thus promising candidates for SHB control. EPNs are small, worm‐like soil invertebrates capable of attacking a wide range of insects. Species of the two EPN families, Steinernematidae and Heterorhabditidae, are associated with symbiotic bacteria (*Xenorhabdus* and *Photorhabdus*, respectively), which are released by the EPN's infective juveniles (IJs) once they enter an insect host. This results in insect septicemia and mortality, thereby enabling the completion of the EPNs' life cycle within the insect cadaver.^43^, ^44^

A few studies have demonstrated the susceptibility of SHB wandering larvae to a limited number of EPNs, including *Heterorhabditis bacteriophora*, *Heterorhabditis indica*, *Heterorhabditis megidis*, *Heterorhabditis zealandica*, *Steinernema carpocapsae* and *Steinernema feltiae*.^36^, ^45^, ^46^, ^47^, ^48^ However, these studies primarily tested only one or a few EPN isolates, and only under laboratory conditions, leaving a key knowledge gap about how EPNs perform in soil under field or semi‐field conditions. The only field study demonstrating SHB susceptibility to EPNs focused on the effect of *H. indica* on SHB emergence rate.^49^ Additionally, research of EPN effects on the pupal stage is scarce^45^ and there is no information about the direct effect of EPNs on SHB adults emerging from the soil.

Soil biota can have both positive and negative effects on EPNs, significantly influencing their efficacy as biological control agents.^50^, ^51^, ^52^ Several predators, such as microarthropods, free‐living nematodes and nematophagous fungi can adversely affect EPN population dynamics.^53^, ^54^, ^55^ Furthermore, fungal extracts can negatively affect the symbiotic bacteria associated with EPNs,^56^, ^57^, ^58^ as well as EPN reproductive rate.^59^ Furthermore, EPNs and entomopathogenic fungi compete for the same resources and it is uncommon for both to co‐infect insect hosts, with one typically dominating the other.^60^ Additionally, soil factors such as organic matter content, texture and mulch cover can influence the tolerance of EPNs to desiccation, affecting their persistence and performance under different environmental conditions.^61^

In this study, we aimed to (1) evaluate the virulence of 32 EPN isolates of five species, *H. bacteriophora*, *H. indica*, *H. zealandica*, *S. carpocapsae* and *S. feltiae*, against wandering larvae, pupae and adults of the SHB in the laboratory, and identify the most susceptible SHB life stages for biological control; (2) based on the laboratory virulence assays, select the most effective EPN isolates, and assess their potential as biocontrol agents against SHB in a semi‐field environment involving mesocosms with natural and autoclaved (sterile) soil. Of these 32 isolates, 28 isolates have recently been isolated from soils across eastern Australia,^62^ while four are commercially available isolates of *H. bacteriophora*, *H. zealandica*, *S. carpocapsae* and *S. feltiae* that were isolated from Australian soils about 40 years ago.^63^

## 2 MATERIALS AND METHODS

### 2.1 Small hive beetle laboratory culture

A SHB laboratory population was established with adults collected from infested honey bee hives on the Hawkesbury campus of Western Sydney University in Richmond (New South Wales) in summer 2021/22. The beetles were maintained in plastic containers (4.5 L; 15 cm depth × 19.5 cm width × 26 cm length) with one mesh‐covered (mesh aperture 160 × 160 μm) ventilation hole (10 cm × 10 cm), kept at 25 ± 2 °C and 60 ± 5% RH in darkness. Adults and larvae were fed pollen dough (renewed every 3 days), prepared by mixing rapeseed pollen, honey and hydrolyzed yeast in a weight ratio of 10:10:1. The mixture was homogenized until a consistent dough‐like texture was achieved. The pollen dough was placed in 90 mm Petri dishes lined with sponges soaked in Milli‐Q water. Every generation, wandering larvae were transferred to sand‐filled containers (14–15% v/w moisture; 7–8 cm deep) for pupation, and misted with a water sprayer every 3–4 days. Upon eclosion, adults were transferred to new plastic containers.

### 2.2 EPN collection, maintenance and storage

We tested 32 isolates belonging to the five EPN species *H. bacteriophora*, *H. indica*, *H. zealandica*, *S. carpocapsae* and *S. feltiae* (Table 1). These included 18 isolates previously isolated from soils across eastern Australia,^62^ 10 isolates newly isolated from soil in Western Sydney and four commercially available isolates provided by Ecogrow (Queanbeyan, NSW; https://ecogrow.com.au/). The 10 newly isolated isolates were baited from soils with *Tenebrio molitor* larvae (mealworms; purchased from Reptile Realm, Brisbane, Queensland) and characterized as per previously published methods^62^ (Figs S1 and S2). The EPNs were subcultured and maintained in the laboratory using *T. molitor* as the host. The *T. molitor* larvae were kept on wheat bran at 25 °C and 70% relative humidity (RH), with carrot slices added on top to maintain moisture in the bran. For EPN subculturing, larvae of *T. molitor* were inoculated with infective juveniles (IJs) and the infected cadavers were individually transferred to a White trap modified as per Kaya and Stock.^64^ After 2 weeks, IJs were harvested and suspended in Ringer's solution (9.0 g NaCl, 0.42 g KCl, 0.37 g CaCl_2_ × 2H_2_O, and 0.2 g NaHCO_3_ dissolved in 1 L distilled water) and stored at 15 °C for 5–7 days before being used in experiments.

**Table 1:** List of evaluated EPN isolates belonging to the genera *Heterorhabditis* and *Steinernema*, collected from localities across eastern Australia. All field‐collected isolates were baited from soil using mealworms (*Tenebrio molitor*), except for the Hi.LMBT, which was baited with Queensland fruit fly (*Bactrocera tryoni*) larvae. Commercial isolates obtained from Ecogrow have the label EG. All isolates except for those with an asterisk (*) have previously been isolated and characterized by Aryal *et al*.^62^, ^73^, ^74^, ^97^ The nine EPN isolates used in the glasshouse experiment are highlighted in bold.

Number	EPN species	Isolate	Coordinates	Collection date
1	*H. bacteriophora*	Hb.HIE1	−33.609965, 150.746438	March 2019
2	*H. bacteriophora*	**Hb.HIE2**	−33.609965, 150.746438	March 2019
3	*H. bacteriophora*	**Hb.EG**	‐	NA
4	*H. indica*	**Hi.ECCH**	−33.616071, 150.754905	March 2019
5	*H. indica*	Hi.HIE2	−33.616071, 150.754905	March 2019
6	*H. indica*	**Hi.HRN**	−23.442316, 151.915879	Oct 2019
7	*H. indica*	Hi.HRN2	−23.442316, 151.915879	Oct 2019
8	*H. indica*	**Hi.LMBT**	−23.906647, 152.393471	Oct 2019
9	*H. indica*	Hi.QF6	−26.703622, 152.949518	Oct 2019
10	*H. indica*	Hi.QFSC6	−26.703622, 152.949518	Oct 2019
11	*H. indica*	Hi.QGL	−25.129154, 151.996016	Oct 2019
12	*H. indica*	Hi.QGLB	−25.129154, 151.996016	Oct 2019
13	*H. indica*	Hi.PO1(6a)*	−33.609470, 150.745377	Jan 2023
14	*Heterorhabditis sp*.	H.PO*	−33.609470, 150.745377	Jan 2023
15	*H. indica*	Hi.PO2*	−33.609470, 150.745377	Jan 2023
16	*H. indica*	Hi.SO1*	−33.609902, 150.746518	Jan 2023
17	*H. indica*	Hi.SO2*	−33.609902, 150.746518	Jan 2023
18	*H. indica*	Hi.TMN8*	−33.609965, 150.746438	Jan 2023
19	*H. indica*	Hi.TM8(B) *	−33.609965, 150.746438	Jan 2023
20	*H. zealandica*	Hz.BB1	−35.725000, 150.172000	Oct 2018
21	*H. zealandica*	Hz.BB2	−35.711306, 150.178111	Oct 2018
22	*H. zealandica*	Hz.BB3	−35.837972, 150.129306	Oct 2018
23	*H. zealandica*	**Hz.NAR1**	−33.392056, 151.332417	Oct 2018
24	*H. zealandica*	**Hz.NAR2**	−33.392056, 151.332417	Oct 2018
25	*H. zealandica*	Hz.NAR3	−33.392056, 151.332417	Oct 2018
26	*H. zealandica*	Hz.NAR4	−33.392056, 151.332417	Oct 2018
27	*H. zealandica*	Hz.EG	‐	NA
28	*S. carpocapsae*	Sc.EG	‐	NA
29	*S. feltiae*	Sf.CPBR2*	−33.611339, 150.749173	Jan 2023
30	*S. feltiae*	Sf.CPB*	−33.611339, 150.749173	Jan 2023
31	*S. feltiae*	Sf.BR1*	−33.824868, 150.946069	Oct 2023
32	*S. feltiae*	Sf.EG	‐	NA

### 2.3 EPN virulence assays with SHB wandering larvae, pupae and adults in the laboratory

The virulence of the 32 EPN isolates was tested in SHB wandering larvae, pupae and adults under laboratory conditions at 25 °C and 70% RH. For EPN bioassays, 50 g sterile washed play sand (Richgro; https://www.bunnings.com.au/) with 12% moisture (w/w) was placed in 90 mm Petri dishes, followed by the addition of 10 wandering larvae. For each EPN isolate, the SHB individuals were inoculated with 100, 200, 500, 1000, 1500 and 2000 IJs/plate delivered in 1 mL Ringer's solution (Table 1). The control received 1 mL Ringer's solution without nematodes. For the larval test, inoculation was performed on the same day, while for the pupal and adult tests, inoculation occurred after 14 days and 27 days, respectively, ensuring that all SHB individuals had reached the required life stage. These time lags were inferred from a preliminary test that showed, under the same experimental conditions, that the wandering larvae develop into pupae in 12–14 days, and into adults in 25–27 days. The plates were incubated at 25 °C and 70% RH and insect mortality was assessed after 7 days. For each EPN isolate, each concentration was tested for three replicated Petri dishes, each with 10 individuals, and this was repeated three times on separate days to validate the results. We confirmed EPN infection by assessing SHB color changes and dissections of the cadavers. The concentration required to kill 50% of the insects (LC_50_) was calculated using Probit analysis of mortality data.^65^

### 2.4 Soil sampling and preparation for the glasshouse experiment

**Figure 1:**
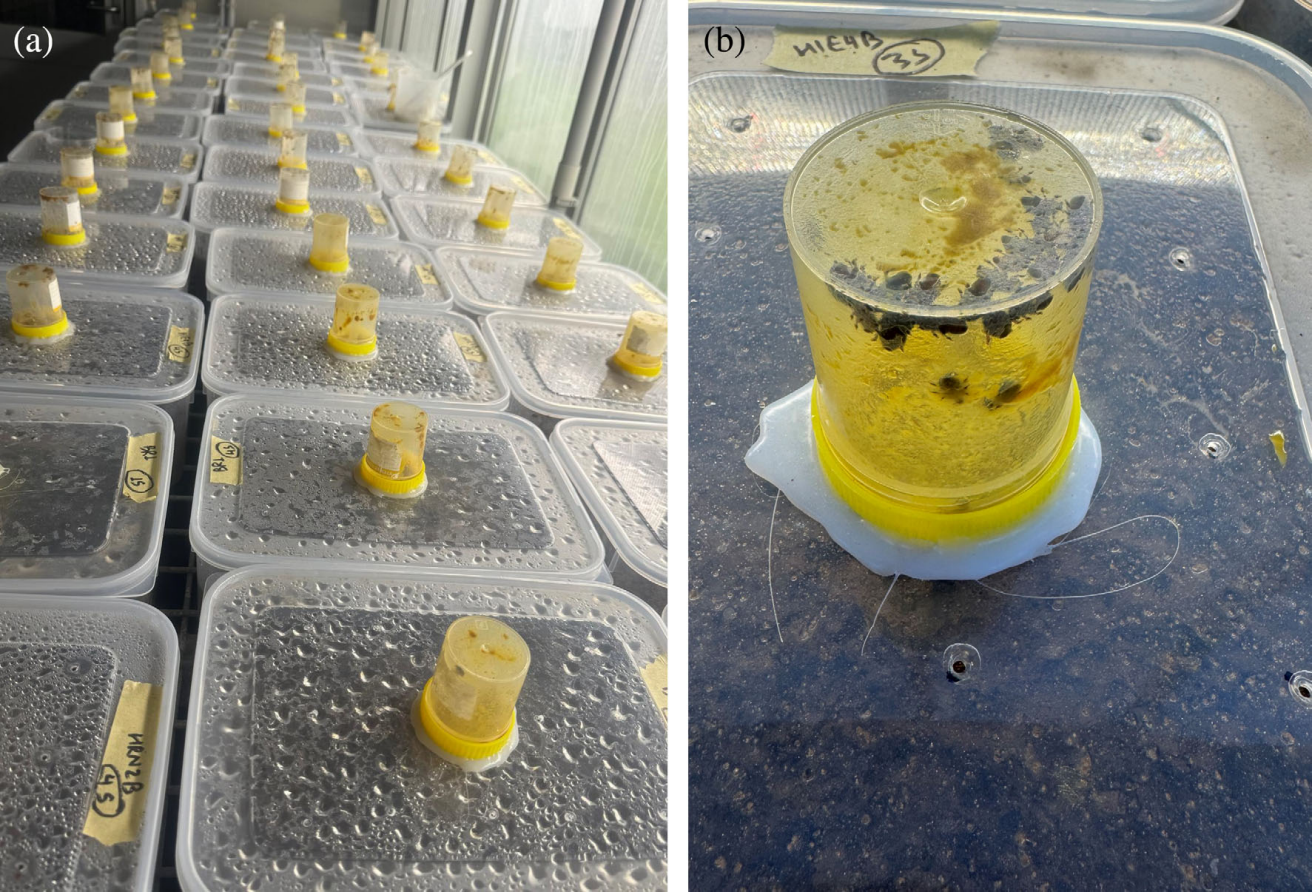
(a) Transparent polypropylene plastic containers fitted with sampling jars on top of the containers. (b) The sampling jars were provided with honey and pollen to attract adult small hive beetles emerging from the soil.

Approximately 300 kg soil was collected from a citrus orchard at the Hawkesbury campus of Western Sydney University. Soil was sampled from underneath five trees across different parts of the orchard, at a depth of 15 cm using a hand shovel. Soil texture (loamy sand), pH (6.2), organic matter (8.9%) and moisture (10.9%) was measured as previously described^62^ (Supplementary Material and Methods S1). Half of the soil was autoclaved (121 °C for 30 min) for sterilization, and then oven‐dried (thereafter referred to as sterile soil); the other half remained untreated (thereafter referred to as natural soil). It is noted that, while this sterilization process would have killed most soil organisms it may also have changed soil parameters,^66^, ^67^ but we ignored such abiotic changes for the purpose of our study. Soil moisture of the sterile soil was adjusted to 10.9% with distilled sterile water to ensure that both the natural and sterile soil had the same moisture content. Briefly, the autoclaved soil was dried completely for 3 days at 100 °C and 10.9 mL Milli‐Q water per 100 g soil was added to obtain the soil moisture level of the sampled natural soil (10.9%). The soil was then transferred to polypropylene plastic containers (12 cm depth × 30 cm width × 22.5 cm length), each containing 3.5 kg soil (approximately 7 cm deep) (Fig. 1(a)).

### 2.5 EPN efficacy against SHB in soil mesocosms in the glasshouse

The efficacy of nine EPN isolates to kill SHB individuals added to the soil containers was tested in a glasshouse chamber at 23.8 ± 2.8 °C and 57.3 ± 3.9% RH. The nine EPN isolates were selected based on their performance in the virulence assays against SHB life stages in the laboratory (Table 1). We included at least one isolate of each EPN species, except for *S. carpocapsae*, which was weak during isolate maintenance. A total of 200 SHB wandering larvae were added to the soil in each container. Soon after all larvae had burrowed into the soil, the soil was inoculated with the concentration of 50 IJs/cm^2^ (33 750 IJs per container with a soil surface area of 675 cm^2^). For each container, IJs were suspended in 10 mL Milli‐Q water and distributed evenly at ten spots (10 × 1 mL). The control received 10 mL Milli‐Q water using the same approach. Five replicates were carried out for each EPN isolate and soil treatment type (natural; sterile).

To ensure ventilation, 12 small holes (smaller than SHB adults) were made in the lid of each container. Each container was equipped with an inverted specimen jar on top, with a tube inserted through the lid and protruding into the specimen jar. This tube allowed SHB adults emerging from the soil to enter the jar, while preventing their return to the soil. The inner side of each specimen jar was coated with a thin layer of organic honey and pollen to serve as an attractant (Fig. 1(b)), and this was renewed every day upon collection of adults. The soil containers of each treatment were evenly distributed on benches in the glasshouse chamber and rotated to different positions every 3 days to reduce the impact of positional effects.

The number of adults that emerged in each container was recorded daily from day 19 (first day of adult emergence) to day 39 after release of wandering larvae. The adult emergence rate was calculated as the proportion of adults that emerged daily relative to the total number of released individuals (*n* = 200) per container. The cumulative emergence rate for each container was then calculated from day 19 to 39. The emerged adults were transferred daily to separate Petri dishes containing moistened filter paper with pollen and honey, and kept at room temperature. Their survival was recorded for the next 7 days. Dead individuals were removed and then dissected to score the proportion of EPN infections in emerging adults as they resurface from the soil.

### 2.6 Data analysis

All data analyses and visualization were performed in R version 4.2.2^68^ using packages agricolae,^69^ dplyr^70^ and ggplot2.^71^ The data were tested for normality using the Shapiro‐Wilkinson test and, as all datasets returned *P*‐values >0.05, the data were further analyzed with linear models. The LC_50_ values and adult emergence and mortality rates were subjected to a one‐way analysis of variance (ANOVA) and then the Tukey's honestly significant difference (HSD) test for multiple comparisons. Furthermore, the EPN‐caused adult mortality was corrected for the control mortality using Abbott's formula^72^:

$$\text{Corrected mortality}=\begin{array}{l} {}[(\%\text{treatment mortality}\\ -\%\text{control mortality})/\left(100-\%\text{control mortality}\right)]\\ \times 100 \end{array}$$

This corrected EPN‐caused mortality accounts for adult beetles that died during the 7 days of incubation after emergence.

## 3 RESULTS

### 3.1 EPN isolates were moderately to highly virulent in wandering larvae, pupae and adults

**Figure 2:**
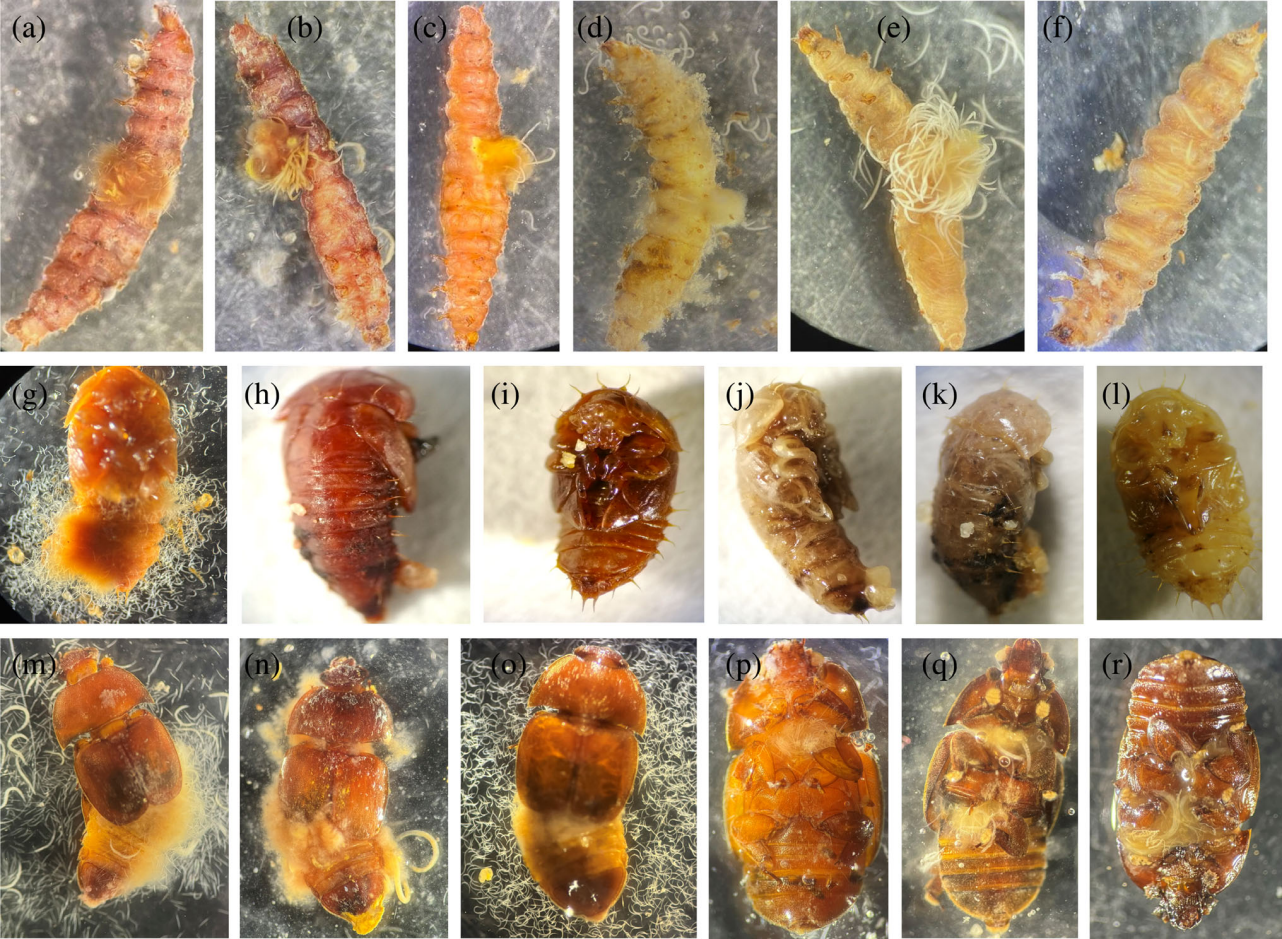
Small hive beetle individuals packed with EPNs: larvae infected with (a) Hb.HIE2, (b) Hi.HRN, (c) Hz.NAR2, (d) Hz.NAR4, (e) Sc.EG and (f) Sf.CPBR2; pupae infected with (g) Hi.HRN, (h) Hb.HIE2, (i) Hz.NAR2, (j) Sc.EG, (k) Hz.BB1 and (l) Sf.CPB; adults infected with (m) Hi.LMBT, (n) Hz.NAR3, (o) Hi.HRN2, (p) Sf.BR1, (q) Sc.EG and (r) Hb.HIE1.

**Figure 3:**
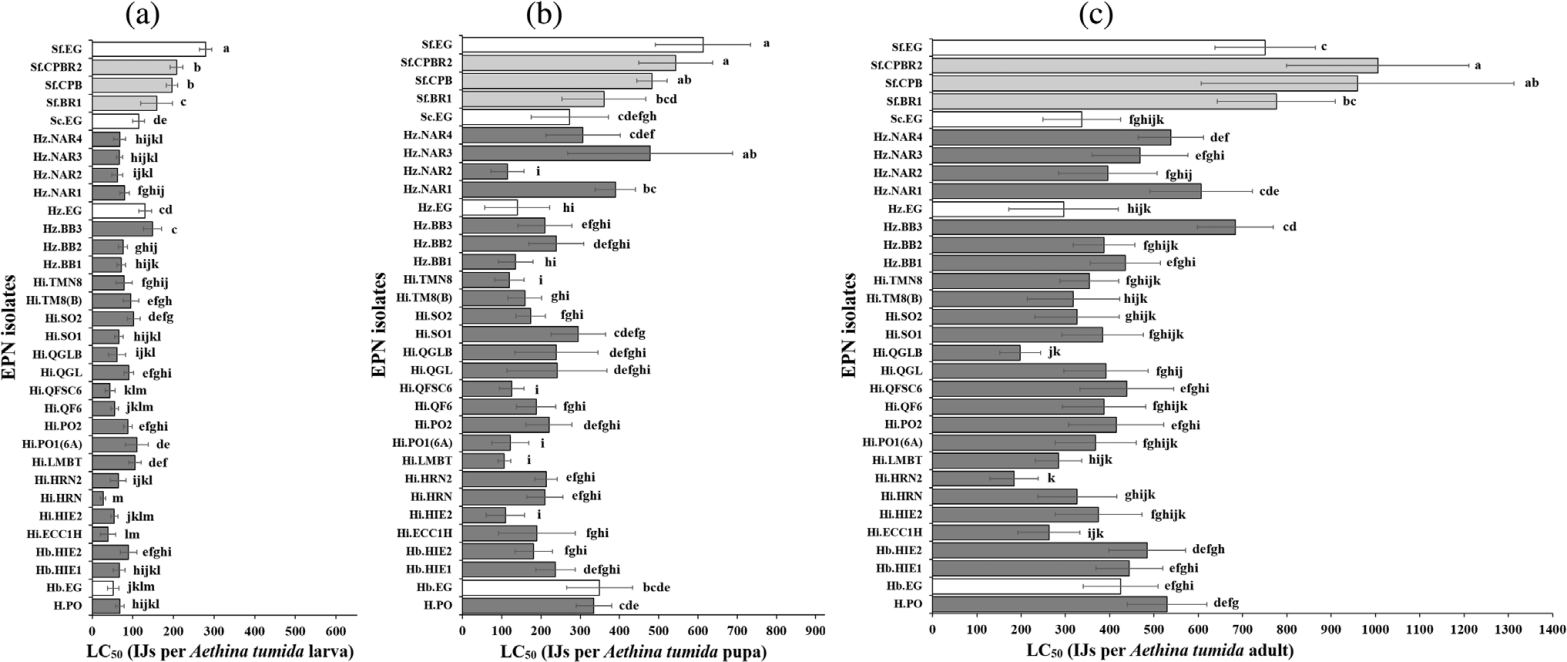
LC_50_ of EPN isolates tested against small hive beetle (a) larvae, (b) pupae and (c) adults after 1 week of exposure to IJs at 25 °C. Error bars indicate SD across nine replicates. Different letters next to the error bars indicate that means are significantly different from each other (Tukey's honestly significant difference test, *P* < 0.05). Lower values indicate higher virulence. The commercially available isolates are represented by white bars, *Steinernema* isolates by grey bars and *Heterorhabditis* isolates by black bars.

All 32 EPN isolates were able to infect SHB wandering larvae, pupae and adults (Fig. 2; Table S1). The mean LC_50_ value ranged from 26.77 ± 6.76 to 278.76 ± 15.08 IJs for wandering larvae (Fig. 3(a)), 106.56 ± 16.09 to 613.07 ± 121.31 IJs for pupae (Fig. 3(b)), and 183.91 ± 54.88 to 1005.31 ± 206.35 IJs for adults (Fig. 3(c)). The isolates with the lowest and highest LC_50_ values were Hi.HRN and Sf.EG for wandering larvae, Hi.LMBT and Sf.EG for pupae, and Hi.HRN2 and Sf.CPBR2 for adults, respectively.

Comparing overall susceptibility to all EPN isolates, mortality varied significantly between SHB larvae and pupae (*F*_63,512_ = 22.7; *P* ≤ 0.0001), pupae and adults (*F*_63,512_ = 26.3; *P* ≤ 0.0001), and larvae and adults (*F*_63,512_ = 70.8; *P* ≤ 0.0001). Significant differences in mortality caused by the different EPN isolates were also observed in wandering larvae (*F*_31,256_ = 95.8; *P* ≤ 0.0001), pupae (*F*_31,256_ = 25.7; *P* ≤ 0.0001) and adults (*F*_31,256_ = 25.9; *P* ≤ 0.0001) (Fig. 3). For *Heterorhabditis*, the mortality caused by the different isolates also varied significantly in wandering larvae (*F*_26,216_ = 68.3; *P* ≤ 0.0001), pupae (*F*_26,216_ = 8.9; *P* ≤ 0.0001) and adults (*F*_26,216_ = 25.9; *P* ≤ 0.0001) (Fig. 3). Similarly, for *Steinernema*, the mortality caused by the different isolates also varied significantly in wandering larvae (*F*_4,40_ = 253.1; *P* ≤ 0.0001), pupae (*F*_4,40_ = 43.1; *P* ≤ 0.0001) and adults (*F*_4,40_ = 32.9; *P* ≤ 0.0001) (Fig. 3).

### 3.2 EPNs reduced SHB emergence from soil and caused mortality in emerged adults

**Figure 4:**
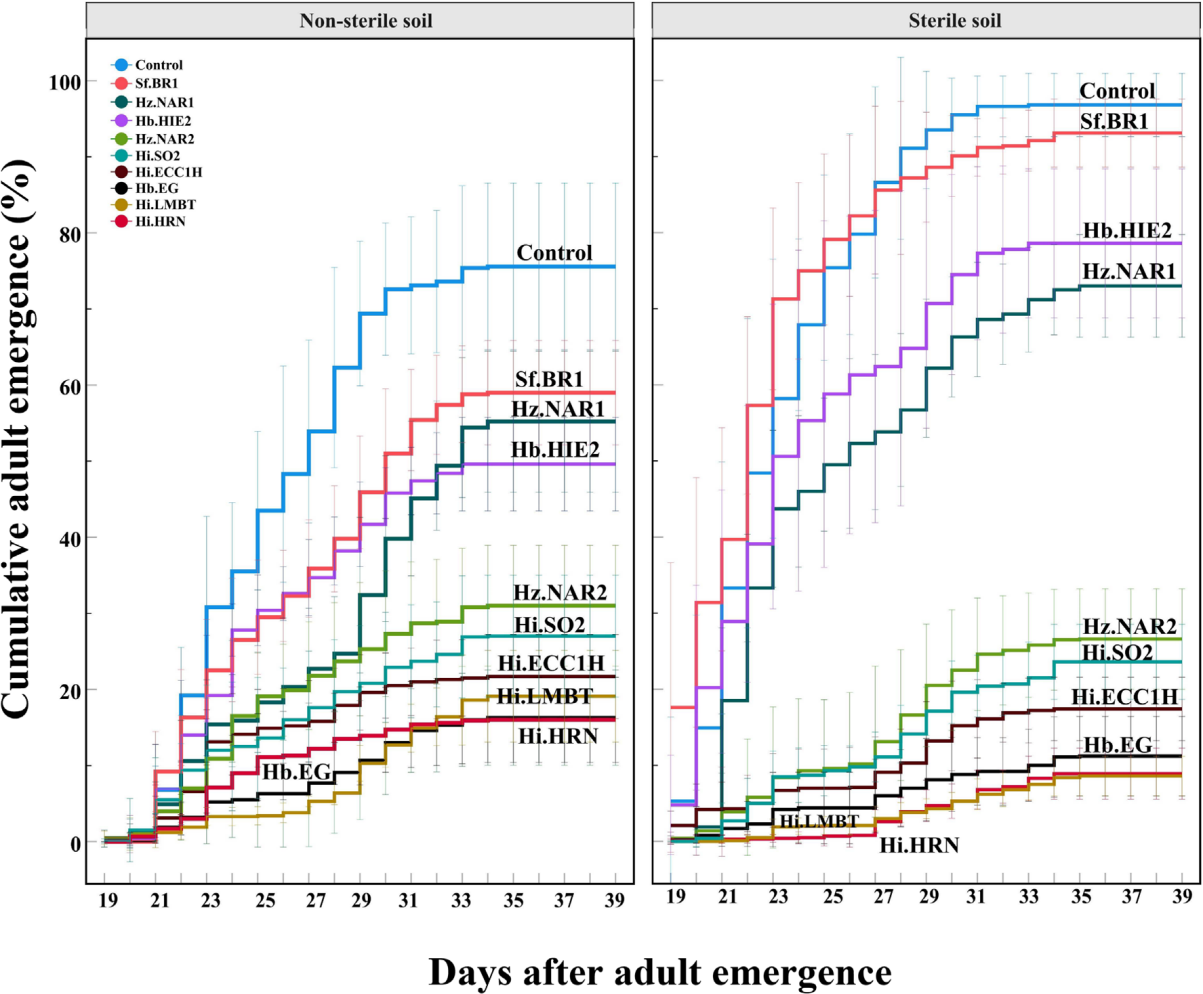
Cumulative adult emergence (%) of small hive beetle over time in natural and sterile soil after EPN treatment. Error bars indicate the standard deviation across five replicates. The first adult emergence was on day 19 after EPN treatment.

**Figure 5:**
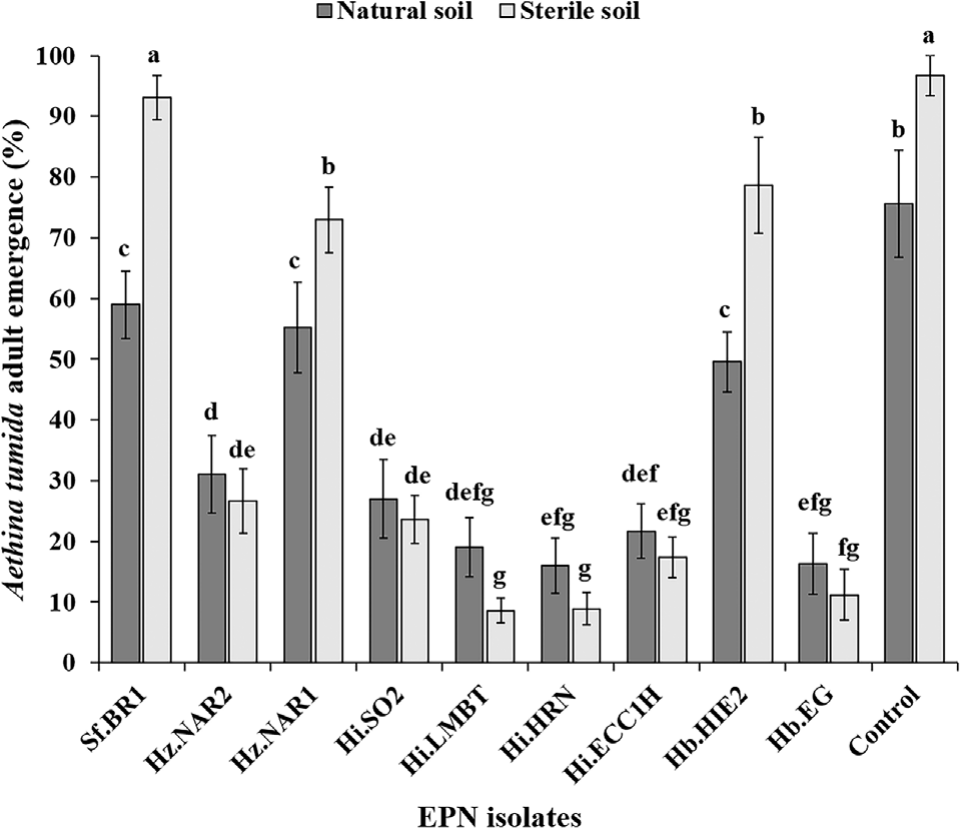
Overall percentage of small hive beetle adults that emerged from EPN‐treated soil in plastic containers. The dark grey sections represent adult emergence in natural soil, and light grey sections represent emergence in sterile soil. Error bars indicate the standard deviation across five replicates. Different letters and numbers on top of the error bars indicate that means are significantly different (Tukey's honestly significant difference test, *P* < 0.05).

All nine EPN isolates significantly reduced SHB adult emergence with notable differences between isolates in natural (*F*_9,40_ = 62.1, *P* < 0.001) and sterile soil (*F*_9,40_ = 338.3, *P* < 0.001), as well as between natural and sterile soil (*F*_19,80_ = 154.6, *P* < 0.001) (Fig. 4) and overall (Fig. 5). In natural soil, the adult SHB emergence ranged from 16 ± 4.5% (Hi.HRN) to 59 ± 5.5% (Sf.BR1), while for sterile soil, it ranged from 8.6 ± 2.1% (Hi.LMBT) to 93.1 ± 3.6% (Sf.BR1) (Fig. 5). In comparison, the control group showed a higher emergence rate of 75.6 ± 8.8% in natural soil and 96.8 ± 3.3% in sterile soil (Fig. 5).

**Figure 6:**
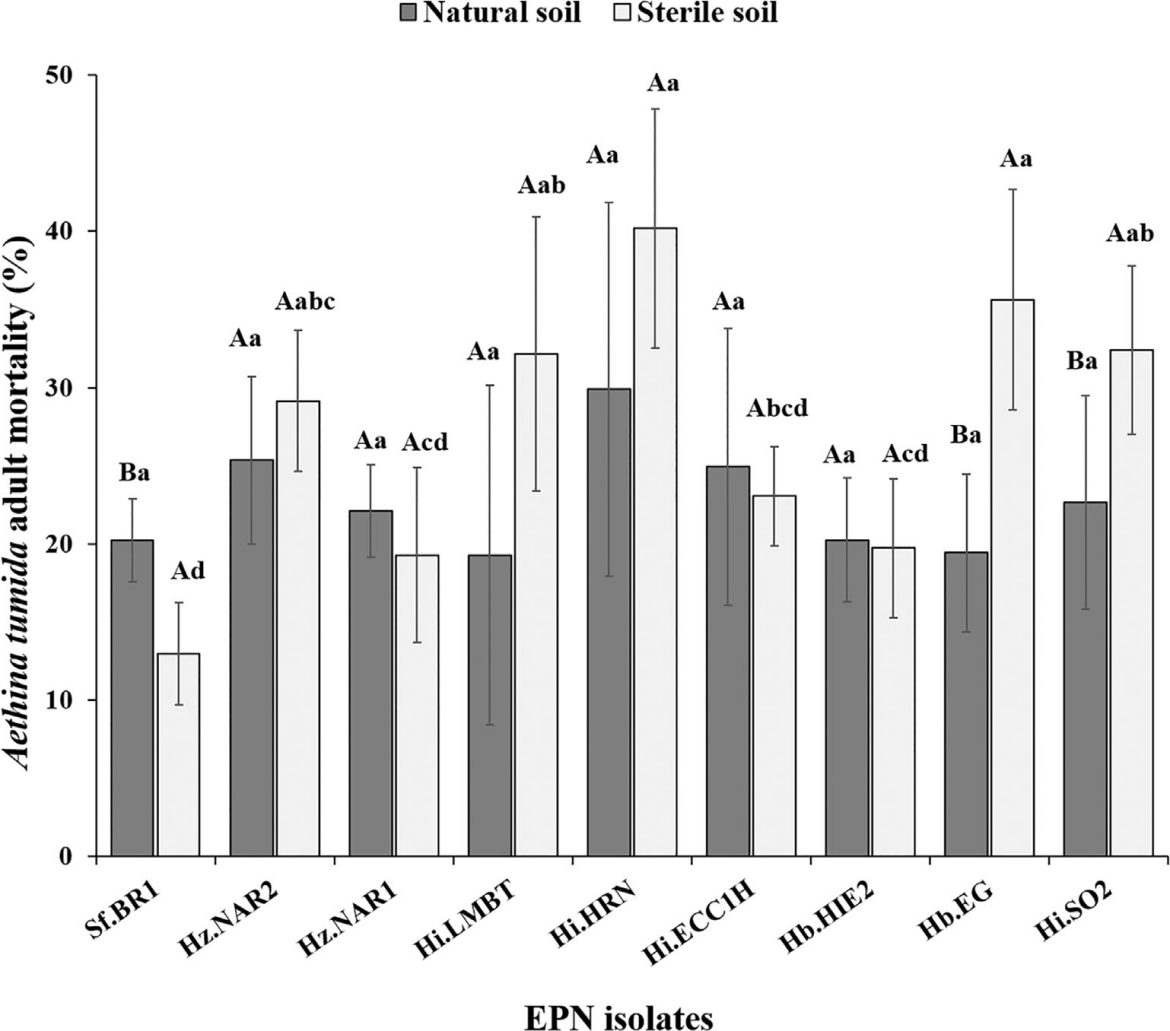
Corrected EPN‐caused adult small hive beetle mortality (%) following their emergence in natural soil (dark grey) and sterile soil (light grey) after EPN treatment. Error bars indicate the standard deviation across five replicates. Different letters next to the error bars indicate that means are significantly different (uppercase letters for comparison between natural and sterile soil within same isolate; lowercase letters for comparison of all EPN isolates within either natural or sterile soil; Tukey's honestly significant difference test, *P* < 0.05).

**Figure 7:**
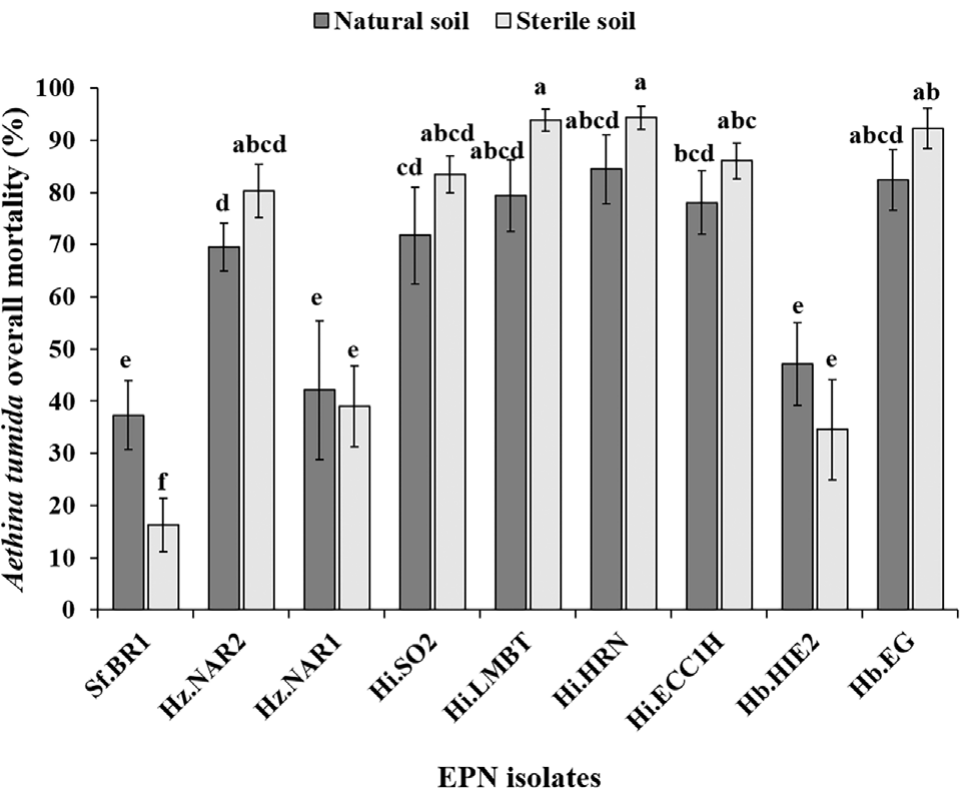
Corrected overall EPN‐caused small hive beetle mortality (%) experienced inside and outside of natural soil (dark grey) and sterile soil (light grey) after EPN treatment of soil. Error bars indicate the standard deviation across five replicates. Different letters next to the error bars indicate that means are significantly different (uppercase letters for comparison between natural and sterile soil within same isolate; lowercase letters for comparison of all EPN isolates within either natural or sterile soil; Tukey's honestly significant difference test, *P* < 0.05).

The corrected EPN‐induced mortality of emerged adult SHB ranged from 20.2 ± 2.7% for Sf.BR1 to 29.9 ± 11.9% for Hi.HRN in natural soil, and 12.9 ± 3.2% for Sf.BR1 to 40.1 ± 7.7% for Hi.HRN in sterile soil (Fig. 6), with significant differences between isolates in sterile soil (F_8,36_ = 11.38, *P* < 0.001), as well as between natural and sterile soil (*F*_17,72_ = 5.54, *P* < 0.001). However, the variation in EPN‐induced adult mortality among isolates was smaller in natural soil than sterile soil; no significant differences in corrected EPN‐induced adult mortality were found among isolates in natural soil (*F*_8,36_ = 1.19, *P* = 0.329) (Fig. 6). Further, the overall corrected EPN‐induced mortality, including the mortality that occurred within and outside soil (after emergence), ranged from 37.4 ± 6.9% for Sf.BR1 to 84.4 ± 6.7% for Hi.HRN in natural soil, and 16 ± 5.1% for Sf.BR1 to 94.4 ± 2.4% for Hi.HRN2B in sterile soil (Fig. 7). However, the corrected EPN‐induced mortality for individual EPN isolates showed no significant differences between natural and sterile soil, except for *S. feltiae* Sf.BR1 (*F*_1,8_ = 31.03, *P* < 0.001) which was the least effective isolate (Fig. 7). However, there were significant differences among corrected mortality for EPN isolates in both natural soil (*F*_8,36_ = 27.9, *P* < 0.001), sterile soil (*F*_8,36_ = 163.7, *P* < 0.001) and between natural and sterile soil (*F*_17,72_ = 66.5, *P* < 0.001) (Fig. 7).

## 4 DISCUSSION

Our study provides the most comprehensive assessment of EPN isolates against SHB to date. We demonstrated that Australian isolates of *H. bacteriophora*, *H. indica*, *H. zealandica*, *S. carpocapsae* and *S. feltiae* were all virulent against multiple SHB life stages. The virulence varied significantly across EPN species and isolates. Additionally, SHB life stages exhibited different susceptibility, with wandering larvae being more susceptible than pupae, and pupae more susceptible than adults. Specifically, *H. indica* (Hi.HRN) was the most virulent EPN isolate against SHB, and wandering larvae were by far the most vulnerable SHB life stage, being about 10 times more susceptible to EPN infection than pupae or adults. This finding is consistent with previous studies on other pest species, such as the Queensland fruit fly (*Bactrocera tryoni*), where *H. indica* (Hi.HRN) was also highly virulent in larvae.^73^, ^74^ Furthermore, our glasshouse experiment demonstrated that EPNs can effectively control SHB populations in soil mesocosms. The *H. indica* isolates Hi.HRN and Hi.LMBT were particularly effective, corroborating previous research by Ellis *et al*.^45^ which showed high mortality rates due to *H. indica* in both SHB larvae and pupae.

While we found that *H. indica* isolates resulted in the highest mortality among the tested isolates against SHB wandering larvae, pupae and adults, their virulence was developmental stage‐specific. For instance, Hi.HRN was most virulent against wandering larvae, Hi.LMBT against pupae, and Hi.HRN2 against adults. Surprisingly, Hi.LMBT exhibited low virulence in wandering larvae, while Hi.HRN2 showed low virulence in pupae. A sand assay by Ellis *et al*.^45^ found *H. zealandica* to be more virulent than *H. bacteriophora* in SHB larvae. In contrast, Spooner‐Hart^48^ reported that a *H. bacteriophora* isolate was more effective than a *H. zealandica* isolate in causing SHB mortality (both isolates from Ecogrow). Interestingly, our study found no significant differences in mortality rates between these two isolates, also from Ecogrow.

A previous study by Cuthbertson *et al*.^36^ that screened several *Steinernema* species and two entomopathogenic fungi (*Beauveria bassiana* and *Lecanicillium muscarium*) against SHB wandering larvae demonstrated that *S. carpocapsae* is more effective than *S. feltiae*, a finding consistent with our results; they furthermore found that *Steinernema* was more effective than the entomopathogenic fungi. Sanchez *et al*.^46^ also found that *S. carpocapsae* was the most effective EPN against SHB in the soil. Cabanillas and Elzen^75^ also reported *S. carpocapsae* causing high larval mortality, although with an LC_50_ of 204 IJs, which is higher than the LC_50_ of 114 IJs found for *S. carpocapsae* in our study. Moreover, we showed that *S. feltiae* was the least virulent EPN species against SHB. This is consistent with the previous laboratory bioassays conducted on filter paper in Australia^48^ and in laboratory assays with three different field‐collected soils in the USA^46^ that concluded that *S. feltiae* was not an efficient biological control agent for SHB.

Ellis *et al*.^45^ previously reported low SHB pupal mortality with *H. megidis* in sterilized soil, observing no significant increase in mortality compared to control groups, and no differences in larval and pupal mortality. In contrast, our results demonstrated that the most virulent isolate in larvae (Hi.HRN) was nearly four times more virulent than the most virulent isolate in pupae (Hi.LMBT), and seven times more virulent than the most virulent isolate in adults (Hi.HRN2). Furthermore, we found significant variation in virulence both within and between EPN species across all developmental life stages of SHB. For example, in wandering larvae, there was a 4‐fold difference in LC_50_ between the most (Hi.HRN2) and least (Hi.LMBT) virulent *H. indica* isolates, a 2‐fold difference between the most (HB.EG) and least (HB.HIE2) virulent *H. bacteriophora* isolates, and a 2‐fold difference between the most (HZ.NAR2) and least (HZ.BB3) virulent *H. zealandica* isolates. These differences may be attributed to differences in host finding, host penetration and infection processes between different isolates of EPN species. For example, *H. indica* isolate Hi.HRN2 showed significantly higher penetration rates than Hi.QF6.^74^

Our glasshouse experiment also showed that the emergence rate of SHB adults varied between the natural and sterile soil control treatments, with reduced emergence rates in natural soil compared to sterile soil in controls without EPN treatment. This is likely due to the presence of naturally occurring EPNs and other entomopathogenic soil microbes in the soil sampled for this experiment contributing to the overall SHB mortality. However, our experiments also showed that soil applications with some EPN isolates reduced adult emergence by >80%, suggesting their potential as an effective biocontrol for SHB in natural field environments. Previous findings by Sanchez *et al*.^46^ identified *S. feltiae* as one of the least virulent isolates against SHB larvae in both natural and sterile soil in a laboratory setting, aligning with our results showing *S. feltiae* Sf.BR1 as the least effective isolate. However, while Sanchez *et al*.^46^ reported *H. indica* and *H. bacteriophora* as the least virulent against SHB larvae across both soil types, our study found that *H. indica* Hi.HRN, Hi.LMBt, and Hi.ECC1H and *H. bacteriophora* Hb.HIE2 were among the most effective isolates in controlling SHB emergence.

EPNs interact with other soil organisms in various beneficial and harmful ways, including predation, infection,^51^, ^76^, ^77^, ^78^ exploitation,^79^, ^80^ competition,^50^, ^78^, ^81^, ^82^, ^83^ phoresy^84^, ^85^ and facilitation.^86^ Some of these interactions may negatively affect the efficiency of EPNs in controlling pests. However, research on these interactions in field settings is limited. Therefore, more studies are needed to better understand how these biotic interactions influence EPN survival and effectiveness in pest control.

Some previous northern hemisphere studies have shown that steinernematids are particularly effective in controlling SHB.^36^, ^46^, ^87^ However, our results show that *H. indica* may be a better choice for warmer climates, as it can tolerate heat and may be better adapted to warmer regions.^62^, ^88^ Commercial EPN products such as NemaSeek™ with *H. indica* are available for pest control (including SHB) in the USA, with a recommended application of 5 million IJs per 20 m^2^ (www.arbico-organics.com). Our semi‐field assay with a concentration of 50 IJs/cm^2^ (approximately double the NemaSeek™ recommended application concentration) showed that EPNs were able to reduce SHB emergence by approximately 84% in natural soil and 93% in sterile soil. In comparison to the generally large soil application area that is required to control many plant pests, EPN soil application against SHB can focus on a soil perimeter of a couple of meters underneath and around a hive. Therefore, the application of a relatively higher concentration of EPNs is unlikely a significant increase in overall cost.

In conclusion, our study represents the most comprehensive effort to date in assessing the virulence of various EPN isolates against SHB in the laboratory, as well as experiments involving natural soil under semi‐field conditions. We demonstrated that EPNs have significant potential to control SHB life stages in soil, particularly the wandering larvae. Among the EPNs, *H. indica* isolates, specifically Hi.HRN2 and Hi.LMBT, emerged as promising candidates as biocontrol agents. The higher virulence of *H. indica* isolates in SHB when compared to other EPN isolates may be due to a complex interplay of several biochemical and physiological factors, such as superior host recognition, more effective penetration, more virulent symbiotic bacteria, higher reproductive fitness and more effective insect immune suppression.^89^, ^90^ Despite the biocontrol potential of EPNs, their impact on honey bee safety requires further investigation. EPNs naturally occur in soil ecosystems, and likely underneath hives. We are not aware of any reports of honey bees that have naturally acquired EPN infections from the soil; hence, EPN soil applications may pose minimal risks to honey bees. However, this requires further investigation, in particular if in‐hive applications of EPNs are considered as there are conflicting reports about EPN effects if directly applied on honey bees.^91^, ^92^

For optimal SHB management, EPNs should be incorporated into an integrated pest management (IPM) strategy, alongside other methods like trapping, regular hive inspections and maintaining proper sanitation. Furthermore, combining EPNs with other microbial agents such as entomopathogenic fungi and bacteria can have synergistic^93^, ^94^, ^95^ or antagonistic effects.^56^ These outcomes depend on factors such as the type of EPN and microbial isolates used, the target pest and the application rate of the entomopathogens.^95^, ^96^ Further research is needed to assess the compatibility and potential synergistic effects of combining EPNs with other microbial agents before incorporating them into IPM programs together. Investigating their compatibility with other IPM strategies in hive settings will be crucial to ensuring a practical and sustainable management approach for beekeepers. Future studies should focus on field trials involving promising isolates like Hi.HRN and Hi.LMBT, to evaluate their efficacy in both soil and hive settings. These trials should also consider the potential risks to honey bee health from in‐hive applications, and focus on evaluating their efficacy under real‐world conditions, including their ability to establish and persist in different soil types, their effectiveness against SHB populations across various environmental conditions and their potential impact on non‐target organisms. Moreover, research should explore optimized application methods, such as timing and formulation, to enhance their field performance. The potential for attract‐and‐kill approaches using traps with SHB attractants should also be investigated. This approach has recently been explored for other pests, such as *B. tryoni*.^97^ Ultimately, a key research question will be how to best apply EPNs in the field settings to maximize the effectiveness in controlling SHB.

## AUTHOR CONTRIBUTIONS

SA, MR and AK conceptualized and designed the experiments. SA performed the experiments, collected and analyzed the data, under the guidance of MR and AK and advice of UN, RSH, CH and JC. SA analyzed the data. SA and MR wrote the manuscript together with AK and with input of all other authors. MR was responsible for research funding.

## Supporting information


**Data S1:** Supporting Information.

## Data Availability

All data are contained within the manuscript and supplementary material, and the raw data have been uploaded to Figshare (https://doi.org/10.6084/m9.figshare.27299922).
