# Supplementary material for: Virulence and biocontrol potential of entomopathogenic nematodes against soil‐dwelling stages of the small hive beetle under laboratory and semi‐field conditions
Source: Pest Manag Sci. 2025 Mar 11;81(7):4004–15. doi: 10.1002/ps.8766 (PMC12159372; doi:10.1002/ps.8766)
Supplement: Supplementary file 1 — Data S1: Supporting Information. [file PS-81-4004-s001.docx]

**Supplementary Method S1**

Soil parameters were assessed following previously published protocols^1^. Soil moisture was calculated by subtracting the dry weight (after oven-drying at 105°C for 48 hours) from the original (wet) weight, expressed as a percentage of the original (wet) weight. To determine soil organic matter (SOM), dry soil was ashed in a muffle furnace at 360°C for 4 hours, and SOM was calculated as the difference between the dry and ashed weights, relative to the dry weight. For pH measurement, 5 g of sieved (2 mm) fresh soil was mixed with 25 ml of Milli-Q water in a 50 ml centrifuge tube and left for two hours before measuring the pH with a SevenCompact S220 pH meter (Mettler-Toledo).

Soil texture was determined using the hydrometer method. First, 25 g of soil was mixed with 25 ml of 5% Calgon solution and 250 ml water. This mixture was then transferred to a 500 ml measuring cylinder and filled to the 500 ml mark. After mixing, a hydrometer reading was taken at 5 minutes (Rt5) to measure the silt and clay content, and another reading was taken at 90 minutes (Rt90) to measure the clay content. A blank reading (R0) for the Calgon-water solution was also taken to correct for the Calgon effects. The temperature (T °C) was recorded, and a correction was applied using the equations below. The results were then plotted on a triangular diagram to determine the soil’s textural class^2^.

Summation % P1 at 5 minutes = [Rt_5_ - R_0_ + (T - 19.5) × 0.3] / (2 × 25) × 100

Summation % P2 at 90 minutes = [(Rt_90_) - R_0_ +(T - 19.5) × 0.3] / (2 × 25) ×100

Sand % = 100 - P1

Clay % = P2

Silt % = P1 - P2

# **Supplementary Figures:**

**Supplementary Figure S1**: Maximum likelihood tree of three *Steinernema feltia* isolates from eastern Australia used in our study (triangles) together with other *Steinernema* species based on the analysis of the D2-D3 segment (434–3493 bp), with *Heterorhabditis zealandica* as an outgroup. Bootstrap values are indicated at the nodes (1000 replicates). GenBank accession numbers are given in parentheses. Scale bar represents number of substitutions per site.

**Supplementary Figure S2:** Maximum likelihood tree of six *Heterorhabditis* isolates from eastern Australia used in our study (triangles) together with other *Heterorhabditis* species based on the analysis of the D2-D3 segment (434–1018 bp), with *Oscheius tipulae* as an outgroup. Bootstrap values are indicated at the nodes (1000 replicates). GenBank accession numbers are given in parentheses. Scale bar represents number of substitutions per site.

**Supplementary Table S1.** LD_50_ values of EPN isolates when tested in small hive beetle larvae, pupae and adults.

| **EPN isolate** | **Replicate #** | **LD_50_** | | |
| --- | --- | --- | --- | --- |
|  |  | **Larvae** | **Pupae** | **Adults** |
| H.PO | 1 | 78 | 393.6 | 546.1 |
| H.PO | 2 | 61.6 | 373.3 | 557.3 |
| H.PO | 3 | 51.8 | 267.4 | 544.6 |
| H.PO | 4 | 56.4 | 304.9 | 530.8 |
| H.PO | 5 | 75.6 | 302.1 | 550.6 |
| H.PO | 6 | 73.6 | 176.7 | 342.1 |
| H.PO | 7 | 65 | 288.9 | 464.1 |
| H.PO | 8 | 82.2 | 224.4 | 546.1 |
| H.PO | 9 | 69.4 | 176.4 | 683.6 |
| H.Po1(6a) | 1 | 126.2 | 56.5 | 471.5 |
| H.Po1(6a) | 2 | 158.3 | 98.9 | 333.5 |
| H.Po1(6a) | 3 | 127.6 | 40.9 | 294.3 |
| H.Po1(6a) | 4 | 128.7 | 140.7 | 294.3 |
| H.Po1(6a) | 5 | 117.9 | 178.2 | 344.5 |
| H.Po1(6a) | 6 | 89.3 | 140.4 | 289.7 |
| H.Po1(6a) | 7 | 81.5 | 158.5 | 559.7 |
| H.Po1(6a) | 8 | 81.9 | 154.8 | 380.9 |
| H.Po1(6a) | 9 | 80.9 | 124.5 | 345.6 |
| H.PO2 | 1 | 81.5 | 229.9 | 466.9 |
| H.PO2 | 2 | 80.3 | 200.4 | 544.6 |
| H.PO2 | 3 | 84 | 140.4 | 565.8 |
| H.PO2 | 4 | 100.2 | 224.8 | 398.9 |
| H.PO2 | 5 | 101.1 | 348.2 | 476.9 |
| H.PO2 | 6 | 91.2 | 200.3 | 342.1 |
| H.PO2 | 7 | 94.7 | 258.1 | 298.4 |
| H.PO2 | 8 | 70.7 | 176.6 | 387.1 |
| H.PO2 | 9 | 87 | 204.1 | 250.9 |
| H.SO1 | 1 | 81.5 | 514.2 | 460.1 |
| H.SO1 | 2 | 57.1 | 298.5 | 348.4 |
| H.SO1 | 3 | 65 | 389.2 | 398.5 |
| H.SO1 | 4 | 67.9 | 224.8 | 470.2 |
| H.SO1 | 5 | 52.2 | 224.8 | 478.8 |
| H.SO1 | 6 | 56.7 | 206.5 | 250.1 |
| H.SO1 | 7 | 80 | 330.9 | 475.1 |
| H.SO1 | 8 | 61.3 | 382.8 | 286.6 |
| H.SO1 | 9 | 73.5 | 259.9 | 292.3 |
| H.SO2 | 1 | 126 | 177.3 | 470.2 |
| H.SO2 | 2 | 87 | 154.2 | 288.3 |
| H.SO2 | 3 | 99.7 | 124.8 | 342.8 |
| H.SO2 | 4 | 81.3 | 202.2 | 288.3 |
| H.SO2 | 5 | 116.7 | 199.3 | 335.7 |
| H.SO2 | 6 | 122.2 | 155.2 | 294.9 |
| H.SO2 | 7 | 99.7 | 232.1 | 464.8 |
| H.SO2 | 8 | 87.8 | 199.4 | 286.9 |
| H.SO2 | 9 | 101.1 | 122.6 | 162.6 |
| H.TM8(B) | 1 | 98.5 | 140.3 | 186.8 |
| H.TM8(B) | 2 | 94.7 | 111.2 | 253.1 |
| H.TM8(B) | 3 | 74 | 110.5 | 287.1 |
| H.TM8(B) | 4 | 129.8 | 224.7 | 406.5 |
| H.TM8(B) | 5 | 81.5 | 139.4 | 472.6 |
| H.TM8(B) | 6 | 109.8 | 200.1 | 282.9 |
| H.TM8(B) | 7 | 115.1 | 203.3 | 464.8 |
| H.TM8(B) | 8 | 81.1 | 123.6 | 289.9 |
| H.TM8(B) | 9 | 74.1 | 176.7 | 219.9 |
| H.TMN8 | 1 | 116.1 | 112.1 | 344.9 |
| H.TMN8 | 2 | 61.3 | 50.7 | 482.1 |
| H.TMN8 | 3 | 93.4 | 78.7 | 287.1 |
| H.TMN8 | 4 | 66.6 | 157.9 | 406.5 |
| H.TMN8 | 5 | 66.5 | 158.6 | 340.5 |
| H.TMN8 | 6 | 88.8 | 111.9 | 409.2 |
| H.TMN8 | 7 | 88.8 | 157.9 | 288.9 |
| H.TMN8 | 8 | 54.6 | 136.6 | 334.9 |
| H.TMN8 | 9 | 72.6 | 110.5 | 292.3 |
| Hb.EG | 1 | 45.8 | 382.8 | 545.8 |
| Hb.EG | 2 | 60 | 383.9 | 459.4 |
| Hb.EG | 3 | 66.5 | 288.9 | 453.2 |
| Hb.EG | 4 | 39.6 | 531.3 | 393.1 |
| Hb.EG | 5 | 35.7 | 254.8 | 453.2 |
| Hb.EG | 6 | 36.7 | 382.7 | 343.6 |
| Hb.EG | 7 | 72.5 | 334.4 | 527.3 |
| Hb.EG | 8 | 66 | 296.8 | 302.1 |
| Hb.EG | 9 | 44.5 | 286.8 | 343.6 |
| Hb.HIE1 | 1 | 74.9 | 265.9 | 453.2 |
| Hb.HIE1 | 2 | 68.5 | 226.1 | 343.9 |
| Hb.HIE1 | 3 | 52.3 | 176.6 | 336.3 |
| Hb.HIE1 | 4 | 52.6 | 344.1 | 404.1 |
| Hb.HIE1 | 5 | 57.1 | 231.9 | 459.9 |
| Hb.HIE1 | 6 | 68.9 | 225.8 | 449.8 |
| Hb.HIE1 | 7 | 98.2 | 229.9 | 547.9 |
| Hb.HIE1 | 8 | 56.9 | 176.4 | 551.9 |
| Hb.HIE1 | 9 | 67.9 | 255.3 | 453.2 |
| Hb.HIE2 | 1 | 107.6 | 99.9 | 649.5 |
| Hb.HIE2 | 2 | 90.7 | 112.2 | 394.2 |
| Hb.HIE2 | 3 | 89.8 | 123.5 | 456.9 |
| Hb.HIE2 | 4 | 118.8 | 347.8 | 470.9 |
| Hb.HIE2 | 5 | 87.8 | 385.6 | 460.7 |
| Hb.HIE2 | 6 | 89.9 | 350.1 | 605.1 |
| Hb.HIE2 | 7 | 98.4 | 255.6 | 472.5 |
| Hb.HIE2 | 8 | 73.8 | 177.5 | 453.2 |
| Hb.HIE2 | 9 | 47.7 | 159.7 | 399.7 |
| Hi.ECC1H | 1 | 68.5 | 136.9 | 296.3 |
| Hi.ECC1H | 2 | 61.9 | 153.6 | 293.7 |
| Hi.ECC1H | 3 | 56.9 | 87.7 | 258.1 |
| Hi.ECC1H | 4 | 19.7 | 142.2 | 350.9 |
| Hi.ECC1H | 5 | 24.5 | 141.8 | 222.3 |
| Hi.ECC1H | 6 | 35.9 | 112.1 | 123.9 |
| Hi.ECC1H | 7 | 33.1 | 295.6 | 258.1 |
| Hi.ECC1H | 8 | 26.9 | 382.2 | 342.9 |
| Hi.ECC1H | 9 | 24.7 | 253.1 | 219.9 |
| Hi.HIE2 | 1 | 65.4 | 140.8 | 348.4 |
| Hi.HIE2 | 2 | 60.5 | 159.6 | 415.6 |
| Hi.HIE2 | 3 | 60 | 143.4 | 220.9 |
| Hi.HIE2 | 4 | 46.7 | 87.7 | 464.8 |
| Hi.HIE2 | 5 | 48.7 | 56.3 | 301.8 |
| Hi.HIE2 | 6 | 40.3 | 50.3 | 405.1 |
| Hi.HIE2 | 7 | 49.4 | 178.2 | 491.2 |
| Hi.HIE2 | 8 | 62.8 | 111.2 | 255.5 |
| Hi.HIE2 | 9 | 56.3 | 56.6 | 470.3 |
| Hi.HRN | 1 | 20.3 | 226.2 | 355.3 |
| Hi.HRN | 2 | 24.6 | 249.1 | 198.1 |
| Hi.HRN | 3 | 22.2 | 176.4 | 282.9 |
| Hi.HRN | 4 | 40.1 | 255.2 | 427.3 |
| Hi.HRN | 5 | 27.2 | 176.6 | 436.9 |
| Hi.HRN | 6 | 18.5 | 220.9 | 198.3 |
| Hi.HRN | 7 | 32.8 | 334.1 | 375.7 |
| Hi.HRN | 8 | 24.9 | 265.9 | 372.4 |
| Hi.HRN | 9 | 30.3 | 194.9 | 291.9 |
| Hi.HRN2 | 1 | 68.2 | 255.4 | 174.9 |
| Hi.HRN2 | 2 | 57.5 | 230.5 | 231.8 |
| Hi.HRN2 | 3 | 47.6 | 219.9 | 197.2 |
| Hi.HRN2 | 4 | 52.9 | 200.4 | 196.8 |
| Hi.HRN2 | 5 | 51.8 | 201.9 | 98.3 |
| Hi.HRN2 | 6 | 47.4 | 196.5 | 176.4 |
| Hi.HRN2 | 7 | 107.7 | 232.1 | 252.1 |
| Hi.HRN2 | 8 | 68.7 | 227.2 | 229.2 |
| Hi.HRN2 | 9 | 74.1 | 156.5 | 98.5 |
| Hi.LMBT | 1 | 105.5 | 100.1 | 296.3 |
| Hi.LMBT | 2 | 136.7 | 88.6 | 290.7 |
| Hi.LMBT | 3 | 105.3 | 88.3 | 252.1 |
| Hi.LMBT | 4 | 93.4 | 125.3 | 388.7 |
| Hi.LMBT | 5 | 115.1 | 109.6 | 330.7 |
| Hi.LMBT | 6 | 99.6 | 123.3 | 256.3 |
| Hi.LMBT | 7 | 98.5 | 125.1 | 295.6 |
| Hi.LMBT | 8 | 84.1 | 111.3 | 228.9 |
| Hi.LMBT | 9 | 112.7 | 87.4 | 222.1 |
| Hi.QF6 | 1 | 62.2 | 199.3 | 452.1 |
| Hi.QF6 | 2 | 43.1 | 198.6 | 458.5 |
| Hi.QF6 | 3 | 43.2 | 122.5 | 290.9 |
| Hi.QF6 | 4 | 62.1 | 628.1 | 513.7 |
| Hi.QF6 | 5 | 56.9 | 595.9 | 449.8 |
| Hi.QF6 | 6 | 56.8 | 440.3 | 439.2 |
| Hi.QF6 | 7 | 67.1 | 254.8 | 344.9 |
| Hi.QF6 | 8 | 62.2 | 250.7 | 283.4 |
| Hi.QF6 | 9 | 43.3 | 224.7 | 255.9 |
| Hi.QFSC6 | 1 | 50.7 | 97.7 | 584.1 |
| Hi.QFSC6 | 2 | 59.8 | 87.6 | 329.5 |
| Hi.QFSC6 | 3 | 65.1 | 109.6 | 387.4 |
| Hi.QFSC6 | 4 | 40.3 | 176.6 | 445.3 |
| Hi.QFSC6 | 5 | 39.4 | 111.3 | 323.3 |
| Hi.QFSC6 | 6 | 28.1 | 154.8 | 378.3 |
| Hi.QFSC6 | 7 | 37.5 | 226.2 | 605.1 |
| Hi.QFSC6 | 8 | 38.3 | 136.6 | 514.8 |
| Hi.QFSC6 | 9 | 37.4 | 155.2 | 376.7 |
| Hi.QGL | 1 | 81.3 | 453.8 | 341.3 |
| Hi.QGL | 2 | 80.9 | 255.4 | 342.2 |
| Hi.QGL | 3 | 75.3 | 382.8 | 288.7 |
| Hi.QGL | 4 | 97.7 | 325.3 | 432.8 |
| Hi.QGL | 5 | 105.4 | 202.2 | 380.6 |
| Hi.QGL | 6 | 95.8 | 226.2 | 449.8 |
| Hi.QGL | 7 | 89.3 | 109.6 | 605.1 |
| Hi.QGL | 8 | 105.6 | 123.6 | 372.9 |
| Hi.QGL | 9 | 80.8 | 88.1 | 313.3 |
| Hi.QGLB | 1 | 68.2 | 177.3 | 175.7 |
| Hi.QGLB | 2 | 89.8 | 177.2 | 124.5 |
| Hi.QGLB | 3 | 74.6 | 125.6 | 178.1 |
| Hi.QGLB | 4 | 39.3 | 335.2 | 175.6 |
| Hi.QGLB | 5 | 38.9 | 260.5 | 251.6 |
| Hi.QGLB | 6 | 44.3 | 158.4 | 197.5 |
| Hi.QGLB | 7 | 62.4 | 466.5 | 283.4 |
| Hi.QGLB | 8 | 89.9 | 226.5 | 196.2 |
| Hi.QGLB | 9 | 42.9 | 228.4 | 197.2 |
| Hz.BB1 | 1 | 82.3 | 100.2 | 344.5 |
| Hz.BB1 | 2 | 74.8 | 110.4 | 339.1 |
| Hz.BB1 | 3 | 56.9 | 78.9 | 403.5 |
| Hz.BB1 | 4 | 82.6 | 222.6 | 544.6 |
| Hz.BB1 | 5 | 74.9 | 155.3 | 476.4 |
| Hz.BB1 | 6 | 62.4 | 177.5 | 459.4 |
| Hz.BB1 | 7 | 82.3 | 138.9 | 540.8 |
| Hz.BB1 | 8 | 74.8 | 124.6 | 451.9 |
| Hz.BB1 | 9 | 56.9 | 109.7 | 357.1 |
| Hz.BB2 | 1 | 67.9 | 177.3 | 388.8 |
| Hz.BB2 | 2 | 81.1 | 175.8 | 340.2 |
| Hz.BB2 | 3 | 68.6 | 226.2 | 347.6 |
| Hz.BB2 | 4 | 89.4 | 261.2 | 458.7 |
| Hz.BB2 | 5 | 62.1 | 254.8 | 470.8 |
| Hz.BB2 | 6 | 62.1 | 157.9 | 413.6 |
| Hz.BB2 | 7 | 89.5 | 224.8 | 347.6 |
| Hz.BB2 | 8 | 81.5 | 383.9 | 457.1 |
| Hz.BB2 | 9 | 81.1 | 289.2 | 261.4 |
| Hz.BB3 | 1 | 149.7 | 155.6 | 625.8 |
| Hz.BB3 | 2 | 163.9 | 177.3 | 763.4 |
| Hz.BB3 | 3 | 143.4 | 122.5 | 554.8 |
| Hz.BB3 | 4 | 184.6 | 228.4 | 775.9 |
| Hz.BB3 | 5 | 170.7 | 232.1 | 625.8 |
| Hz.BB3 | 6 | 145.5 | 199.3 | 775.9 |
| Hz.BB3 | 7 | 128.4 | 351.1 | 762.8 |
| Hz.BB3 | 8 | 138.1 | 262.6 | 648.1 |
| Hz.BB3 | 9 | 111.3 | 157.9 | 616.9 |
| Hz.EG | 1 | 119.9 | 56.7 | 458.4 |
| Hz.EG | 2 | 114.3 | 71.2 | 547.9 |
| Hz.EG | 3 | 115.1 | 56.6 | 264.9 |
| Hz.EG | 4 | 138.8 | 229.9 | 260.5 |
| Hz.EG | 5 | 160.8 | 235.9 | 201.7 |
| Hz.EG | 6 | 122.7 | 255.4 | 200.1 |
| Hz.EG | 7 | 139.5 | 159.2 | 293.3 |
| Hz.EG | 8 | 122.2 | 110.3 | 230.1 |
| Hz.EG | 9 | 139.4 | 79.6 | 207.8 |
| Hz.NAR1 | 1 | 98.3 | 628.1 | 648.1 |
| Hz.NAR1 | 2 | 89.1 | 506.7 | 563.6 |
| Hz.NAR1 | 3 | 61.7 | 428.3 | 555.9 |
| Hz.NAR1 | 4 | 74.9 | 736.3 | 659.6 |
| Hz.NAR1 | 5 | 74.2 | 428.9 | 477.9 |
| Hz.NAR1 | 6 | 81.1 | 382.8 | 465.6 |
| Hz.NAR1 | 7 | 81.1 | 333.1 | 775.9 |
| Hz.NAR1 | 8 | 88.7 | 441.8 | 775.9 |
| Hz.NAR1 | 9 | 67.9 | 289.2 | 536.1 |
| Hz.NAR2 | 1 | 75.2 | 111.9 | 558.8 |
| Hz.NAR2 | 2 | 68.7 | 110.3 | 345.9 |
| Hz.NAR2 | 3 | 61.8 | 111.2 | 343.1 |
| Hz.NAR2 | 4 | 52.4 | 99.5 | 473.8 |
| Hz.NAR2 | 5 | 62.4 | 78.6 | 559.7 |
| Hz.NAR2 | 6 | 36.3 | 50.7 | 349.4 |
| Hz.NAR2 | 7 | 82.2 | 123.6 | 391.8 |
| Hz.NAR2 | 8 | 57.1 | 203.1 | 251.8 |
| Hz.NAR2 | 9 | 61.9 | 139.5 | 290.3 |
| Hz.NAR3 | 1 | 70.9 | 531.3 | 555.9 |
| Hz.NAR3 | 2 | 74.9 | 855.9 | 531.3 |
| Hz.NAR3 | 3 | 75.6 | 623.6 | 398.1 |
| Hz.NAR3 | 4 | 64.7 | 158.4 | 648.1 |
| Hz.NAR3 | 5 | 74.2 | 159.1 | 404.7 |
| Hz.NAR3 | 6 | 57.1 | 140.4 | 293.1 |
| Hz.NAR3 | 7 | 61.8 | 399.6 | 530.2 |
| Hz.NAR3 | 8 | 68.7 | 388.1 | 395.2 |
| Hz.NAR3 | 9 | 57.1 | 292.7 | 459.4 |
| Hz.NAR4 | 1 | 68.6 | 617.1 | 547.9 |
| Hz.NAR4 | 2 | 62.3 | 597.3 | 452.5 |
| Hz.NAR4 | 3 | 52.4 | 328.1 | 477.2 |
| Hz.NAR4 | 4 | 98.1 | 388.1 | 537.1 |
| Hz.NAR4 | 5 | 67.9 | 224.4 | 538.2 |
| Hz.NAR4 | 6 | 63.2 | 200.4 | 528.9 |
| Hz.NAR4 | 7 | 80.4 | 258.2 | 659.6 |
| Hz.NAR4 | 8 | 51.9 | 177.3 | 645.1 |
| Hz.NAR4 | 9 | 62.6 | 346.4 | 459.4 |
| S.BR1 | 1 | 171.74 | 296.1 | 812.9 |
| S.BR1 | 2 | 219.5 | 258.2 | 635.1 |
| S.BR1 | 3 | 131.8 | 288.4 | 935.9 |
| S.BR1 | 4 | 224.3 | 428.9 | 890.1 |
| S.BR1 | 5 | 149.1 | 396.6 | 627.3 |
| S.BR1 | 6 | 134.77 | 374.9 | 605.1 |
| S.BR1 | 7 | 132.92 | 265.3 | 935.9 |
| S.BR1 | 8 | 118.14 | 339.6 | 825.9 |
| S.BR1 | 9 | 145.75 | 123.5 | 718.9 |
| S.CPB | 1 | 221.5 | 507.7 | 896.3 |
| S.CPB | 2 | 182.9 | 504.8 | **1780.8** |
| S.CPB | 3 | 208.7 | 434.5 | 726.1 |
| S.CPB | 4 | 183.4 | 514.2 | 727.5 |
| S.CPB | 5 | 179.1 | 457.6 | 1101.7 |
| S.CPB | 6 | 206.9 | 428.3 | 607.3 |
| S.CPB | 7 | 188.4 | 453.8 | 750.6 |
| S.CPB | 8 | 201.5 | 327.3 | 1109.5 |
| S.CPB | 9 | 193.6 | 336.6 | 935.8 |
| S.CPBR2 | 1 | 202.3 | 440.4 | 908.2 |
| S.CPBR2 | 2 | 213.9 | 396.6 | 1147.3 |
| S.CPBR2 | 3 | 188 | 382.7 | 775.9 |
| S.CPBR2 | 4 | 206.3 | 683.9 | 934.9 |
| S.CPBR2 | 5 | 243.2 | 387.4 | 1065.6 |
| S.CPBR2 | 6 | 208.7 | 514.2 | 759.3 |
| S.CPBR2 | 7 | 206 | 434.7 | 1147.3 |
| S.CPBR2 | 8 | 192 | 506.7 | 905.1 |
| S.CPBR2 | 9 | 206 | 399.6 | 1404.2 |
| Sc.EG | 1 | 109.2 | 179.7 | 290.9 |
| Sc.EG | 2 | 103.8 | 157.9 | 346.2 |
| Sc.EG | 3 | 109.2 | 177.3 | 333.7 |
| Sc.EG | 4 | 103 | 397.8 | 530.4 |
| Sc.EG | 5 | 116 | 177.3 | 407.8 |
| Sc.EG | 6 | 93 | 125.3 | 293.9 |
| Sc.EG | 7 | 129.1 | 870.1 | 306.3 |
| Sc.EG | 8 | 129 | 529.2 | 299.3 |
| Sc.EG | 9 | 136.4 | 595.9 | 222.8 |
| Sf.EG | 1 | 308.1 | 587.4 | 908.2 |
| Sf.EG | 2 | 281.2 | 496.7 | 638.1 |
| Sf.EG | 3 | 271.8 | 617.2 | 775.9 |
| Sf.EG | 4 | 260.1 | 504.8 | 913.9 |
| Sf.EG | 5 | 292.5 | 513.3 | 638.1 |
| Sf.EG | 6 | 276.6 | 381.2 | 649.4 |
| Sf.EG | 7 | 260.1 | 595.9 | 644.8 |
| Sf.EG | 8 | 281.8 | 325.3 | 808.7 |
| Sf.EG | 9 | 276.6 | 433.1 | 778.5 |

**REFERENCES**

1. Aryal, S., Nielsen, U.N., Sumaya, N.H., De Faveri, S., Wilson, C., Riegler, M. Isolation and molecular characterization of five entomopathogenic nematode species and their bacterial symbionts from eastern Australia. Biocontrol 67, 63-74 (2022).
2. McDonald, R. C., Isbell, R. F., Speight, J. G., Walker, J., & Hopkins, M. S. (1998). Australian soil and land survey: field handbook (No. Ed. 2, pp. xv+-190) (1998).
